# Loss of Rbfox1 Does Not Affect Survival of Retinal Ganglion Cells Injured by Optic Nerve Crush

**DOI:** 10.3389/fnins.2021.687690

**Published:** 2021-05-24

**Authors:** Lei Gu, Jacky M. Kwong, Joseph Caprioli, Natik Piri

**Affiliations:** ^1^Stein Eye Institute, University of California, Los Angeles, Los Angeles, CA, United States; ^2^Brain Research Institute, University of California, Los Angeles, Los Angeles, CA, United States

**Keywords:** Rbfox1, retina, ganglion cells, amacrine cells, optic nerve, optic nerve crush

## Abstract

Rbfox1 is a multifunctional RNA binding protein that regulates alternative splicing, transcription, mRNA stability and translation. Its roles in neurogenesis and neuronal functions are well established. Recent studies also implicate Rbfox1 in the regulation of gene networks that support cell survival during stress. We have earlier characterized the expression of Rbfox1 in amacrine and retinal ganglion cells (RGCs) and showed that deletion of *Rbfox1* in adult animals results in depth perception deficiency. The current study investigates the effect of Rbfox1 downregulation on survival of RGCs injured by optic nerve crush (ONC). Seven days after ONC, animals sustained severe degeneration of RGC axons in the optic nerve and significant loss of RGC somas. Semi-quantitative grading of optic nerve damage in control + ONC, control + tamoxifen + ONC, and *Rbfox1*^–/–^ + ONC groups ranged from 4.6 to 4.8 on a scale of 1 (normal; no degenerated axons were noted) to 5 (total degeneration; all axons showed degenerated organelles, axonal content, and myelin sheath), indicating a severe degeneration. Among these three ONC groups, no statistical significance was observed when any two groups were compared. The number of RGC somas were quantitatively analyzed in superior, inferior, nasal and temporal retinal quadrants at 0.5, 1, and 1.5 mm from the center of the optic disc. The average RGC densities (cells/mm^2^) were: control 6,438 ± 1,203; control + ONC 2,779 ± 573; control + tamoxifen 6,163 ± 861; control + tamoxifen + ONC 2,573 ± 555; *Rbfox1*^–/–^ 6,437 ± 893; and *Rbfox1*^–/–^ + ONC 2,537 ± 526. The RGC loss in control + ONC, control + tamoxifen + ONC and *Rbfox1*^–/–^ + ONC was 57% (*P* = 1.44954E-42), 58% (*P* = 1.37543E-57) and 61% (*P* = 5.552E-59) compared to RGC numbers in the relevant uninjured groups, respectively. No statistically significant difference was observed between any two groups of uninjured animals or between any two ONC groups. Our data indicate that Rbfox1-mediated pathways have no effect on survival of RGCs injured by ONC.

## Introduction

The RNA binding protein, fox-1 (Rbfox) homolog family includes three evolutionarily conserved multifunctional proteins, Rbfox1, Rbfox2, and Rbfox3, that regulate RNA metabolism, including alternative splicing, transcription, mRNA stability and translation efficiency. The Rbfox proteins are master regulators of gene networks involved in both neurogenesis and mature neuronal functions. Disruption of Rbfox functions have been associated with several neurodevelopmental and neuropsychiatric disorders, including autism spectrum disorder (ASD), intellectual disability, epilepsy, ADHD, bipolar disorder, schizoaffective disorder and schizophrenia, sleep latency and heart disease (Bhalla et al., 2004; Martin et al., 2007; Sebat et al., 2007; Lal et al., 2013; Amin et al., 2016; McKean et al., 2016; Misra et al., 2020). The functional diversity of these proteins is supported by the expression of a number of isoforms for each family member (Kuroyanagi, 2009; Wei et al., 2016; Conboy, 2017).

Recently we have analyzed the expression of Rbfox1 in adult and differentiating mouse retinas and the effects of downregulation of this protein on visual function and the retinal transcriptome (Gu et al., 2018). We showed that in both developing and mature retinas, Rbfox1 expression is restricted to retinal ganglion cells (RGCs) and amacrine cells (ACs). RGCs are the projection neurons of the retina; they receive visual information from photoreceptors via bipolar (BP) and ACs, process this information and convey it via their axons in the optic nerve to postsynaptic targets in the brain. More than 46 brain regions have been identified in the mouse brain that receive image-forming and non-image visual information from different RGC types (Morin and Studholme, 2014). Functional and genetic classification of mouse RGCs identified at least 32 and 40 groups of RGCs, respectively (Baden et al., 2016; Rheaume et al., 2018). ACs are retinal interneurons that form synaptic connections with BPs and RGCs in the inner plexiform layer (IPL) and are involved in shaping spatial and temporal characteristics of RGC receptive fields (Diamond, 2017). In the mammalian retina, ACs similar to RGCs are represented by more than 30 morphological and functional subtypes. Downregulation of *Rbfox1* in adult animals had no effect on retinal architecture or retinal cell morphology (Gu et al., 2018). However, *Rbfox1* KO mice showed depth perception deficit, suggesting the involvement of Rbfox1 in the regulation of genes that support the functional integrity of the retino-geniculo-cortical pathway. Interestingly, deletion of *Rbfox2* also resulted in depth perception abnormalities, with the normal gross retinal morphology (Gu et al., 2020). Furthermore, the deletion of the *Rbfox3*, which is normally expressed in most types of RGCs, some types of ACs and HCs, had no significant effect on retinal morphology, pupillary light response (PLR) and the optomotor response (Lin et al., 2018). Although, each Rbfox member has been shown to have specific roles in neuronal development, as well as in mature neuron functions (Lee et al., 2009, 2016; Gehman et al., 2011, 2012; Fogel et al., 2012; Hamada et al., 2015, 2016; Jacko et al., 2018; Vuong et al., 2018), the fact that these RNA binding proteins recognize the same (*U)GCAUG* element within their target genes and that many single KO models show no cellular phenotypes and have relatively modest change in the transcriptome than expected suggest possible redundancy in their function. Pan-neuronal *Rbfox1/Rbfox2* double null mice (*Rbfox1^*loxP/loxP*^/Rbfox2^*loxP/loxP*^/Nestin-Cre*^+/–^) on the other hand, exhibit a much more severe phenotype than either single KO and die prenatally (Gehman et al., 2012). This shows the importance of Rbfox1 and Rbfox2 in neurogenesis and neuronal function; even if there is some overlapping in the function of Rbfox proteins, these proteins cannot be considered fully redundant as Rbfox3, for instance, in *Rbfox1/Rbfox2* double KO failed to substitute the missing family members.

One of the recently characterized functions of Rbfox1 is its involvement in post-transcriptional regulation of gene expression in response to stress. Using Drosophila oogenesis as an *in vivo* system for the stress response, it has been shown that Rbfox1 upregulation mediated by stress-responsive miR-980 promotes cell survival (Kucherenko and Shcherbata, 2018). Furthermore, Rbfox1 has been implicated in neuroprotective effect of miR-132 against amyloid β-peptide (Aβ) and glutamate excitotoxicity in Alzheimer’s disease (El Fatimy et al., 2018). Based on these observations, we hypothesize that retinal cells that normally express Rbfox1, such as RGCs, will be more susceptible to the stress and damage in the *Rbfox1* KO animals compared with the wild-type. The current study evaluates the effect of *Rbfox1* downregulation on the survival of RGCs in response to optic nerve crush (ONC), an established procedure for acute injury of RGC axons, which leads to severe and specific degeneration of RGC axons and somata.

## Experimental Procedures

### Generation of Rbfox1 KO Animals

The use of animals and all experimental procedures with animals were approved by the Animal Research Committee of the University of California at Los Angeles and were performed in compliance with the National Institutes of Health Guide for the Care and Use of Animals and the ARVO (The Association for Research in Vision and Ophthalmology) Statement for the Use of Animals in Ophthalmic and Vision Research. Animals were housed in a 12-h light-dark cycle with food and water available *ad libitum*. *Rbfox1* KO animals were generated as described earlier (Gu et al., 2018). Briefly, homozygous transgenic mice with loxP sites flanking *Rbfox1* gene exons 11–12 (*Rbfox1*^*fl/fl*^; kindly provided by Dr. Douglas Black, UCLA; Gehman et al., 2011) were crossed with Tg(UBC-Cre/ERT2)1Ejb mice (Jackson Laboratory, Bar Harbor, ME; Ruzankina et al., 2007) and the resulting heterozygous *Rbfox1*^*fl*/+;^ UBC-Cre^+/–^ mice were crossed to *Rbfox1*^*fl/fl*^ mice to obtain homozygous *Rbfox1*^*fl/fl*^/UBC-Cre^+/–^ animals. The expression of tamoxifen-inducible Cre recombinase gene in UBC-Cre/ERT2 mice is controlled by the human ubiquitin C (UBC) promoter. Cre activity in homozygous *Rbfox1*^*fl/fl*^/UBC-Cre^+/–^ animals was induced with tamoxifen. Tamoxifen (Sigma, St. Louis, MO) was dissolved in corn oil to a final concentration of 50 mg/ml. *Rbfox1*^*fl/fl*^/UBC-Cre^+/–^ and age-matched heterozygous Rbfox1^*fl/*+^ control mice, were administered 200 mg/kg of tamoxifen solution or corn oil (vehicle) every 24 h, for a total of 5 doses by oral gavage.

### Immunohistochemistry

Retinal sections were incubated with blocking solution (20% fetal calf serum, 5% goat serum, 0.1% Triton X-100 in PBS) for 30 min and then with primary antibodies at 4°C overnight. The following primary antibodies were used: anti-Rbfox1 produced in mouse, 1:200 (Novus Biologicals, Littleton, CO); anti-Rbpms produced in rabbit, 1:500 (Kwong et al., 2010); anti-calbindin D-28K produced in rabbit, 1:500 (EMD Millipore, Billerica, MA); anti-calbindin D-28K produced in rabbit, 1:500 (C2724, Sigma). After washing with 0.1% Triton X-100 in PBS, sections were stained with secondary antibodies for 1 h at room temperature. The following secondary antibodies were used: Alexa Fluor 488-conjugated goat anti-rabbit IgG, 1:500; Alexa Fluor 568-conjugated goat anti-mouse IgG, 1:500; and Alexa Fluor 568-conjugated goat-anti-guinea pig IgG, 1:500 (Thermo Fisher Scientific, Canoga Park, CA). Sections were mounted with mounting medium containing DAPI and imaged with a confocal laser scanning microscope Olympus FV3000 (Olympus, MA).

### ONC and Optic Nerve Injury Grading

ONC was performed as described earlier (Wang et al., 2015). Briefly, a conjunctival incision was made on the temporal side of the globe and the optic nerve was exposed without damage to the optic nerve blood supply. Crush was applied approximately 2 mm behind the globe for 2 s with self-closing forceps.

To quantify the axonal injury, an established method of grading optic nerve injury was adopted (Jia et al., 2000; Ishii et al., 2003). The tissues were dissected, fixed, processed, and embedded in acrylic resin. One-micrometer-thick sections of the proximal optic nerve were cut and stained with 1% toluidine blue. The samples were examined under microscope and the optic nerve injury was assessed in a masked fashion using a graded scale ranging from 1 (normal; no degenerated axons were noted) to 5 (total degeneration; all axons showed degenerated organelles, axonal content, and myelin sheath). In total, 6 animals per group were included in the assessment of axonal injury.

### Cell Quantification

RGC quantification was performed on retinal flat mounts as described earlier. The retinas were fixed in 4% paraformaldehyde in 0.1 M phosphate buffer, incubated with 10% serum for 1 h to reduce non-specific staining and then with anti-Rbpms overnight at 4°C. After washing, retinas were incubated with the corresponding secondary antibody overnight at 4°C. The retinas were placed flat with the GCL facing upward. With several radial cuts, the retina was divided into four quadrants: superior, inferior, nasal and temporal and mounted flat on the glass slide with the GCL facing upward. Four sampling fields (0.24 × 0.24 mm each) were imaged at 0.5, 1, and 1.5 mm from the center of optic nerve disc in each retinal quadrant with a confocal laser scanning microscopy (Olympus FV3000, MA). Retinas from six animals per group were used in these experiments. Quantification was performed in a masked manner. Data are presented as the mean ± SD.

### Statistical Analysis

Data are presented as mean ± SD. An unpaired Student’s *t*-test was used for quantitative analysis of axonal damage and RGC densities. *P* < 0.05 was considered statistically significant.

## Results

### Rbfox1 Downregulation Has No Effect on Retinal Morphology in Uninjured or ONC Animals

**FIGURE 1:**
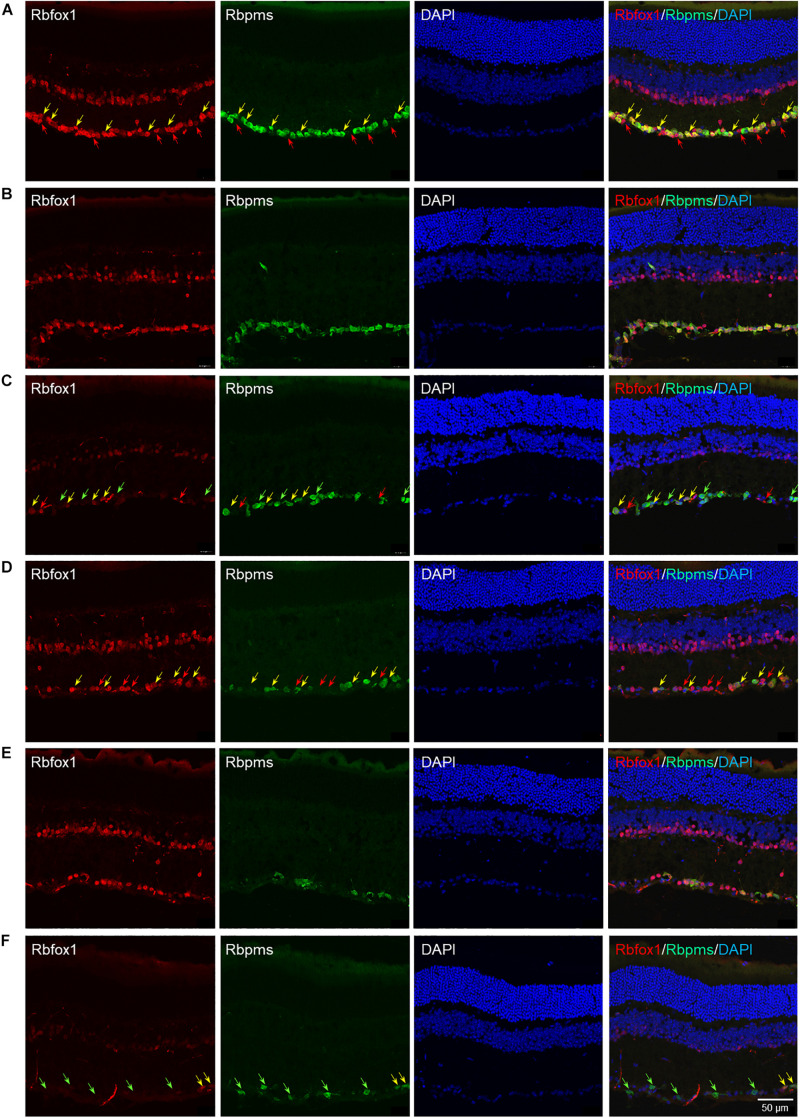
Rbfox1 colocalization with Rbpms-labeled RGCs in uninjured and ONC mouse retinas. In control **(A)** and control + tamoxifen **(B)** retinas, Rbfox1 expression is localized to the GCL and innermost layer of INL. **(C)** Very few Rbfox1-positive cells were present in the GCL of *Rbfox1*^–/–^ animals. **(D–F)** ONC resulted in significant loss of Rbpms-positive cells in control and *Rbfox1*^–/–^ retinas. Some RGCs co-expressing Rbfox1 and Rbpms are pointed by yellow arrows. Rbfox1-positive/Rbpms-negative cells in the GCL are pointed with red arrows. Green arrows point at Rbpms-positive/Rbfox1-negative RGCs in *Rbfox1*^–/–^ retinas.

**FIGURE 2:**
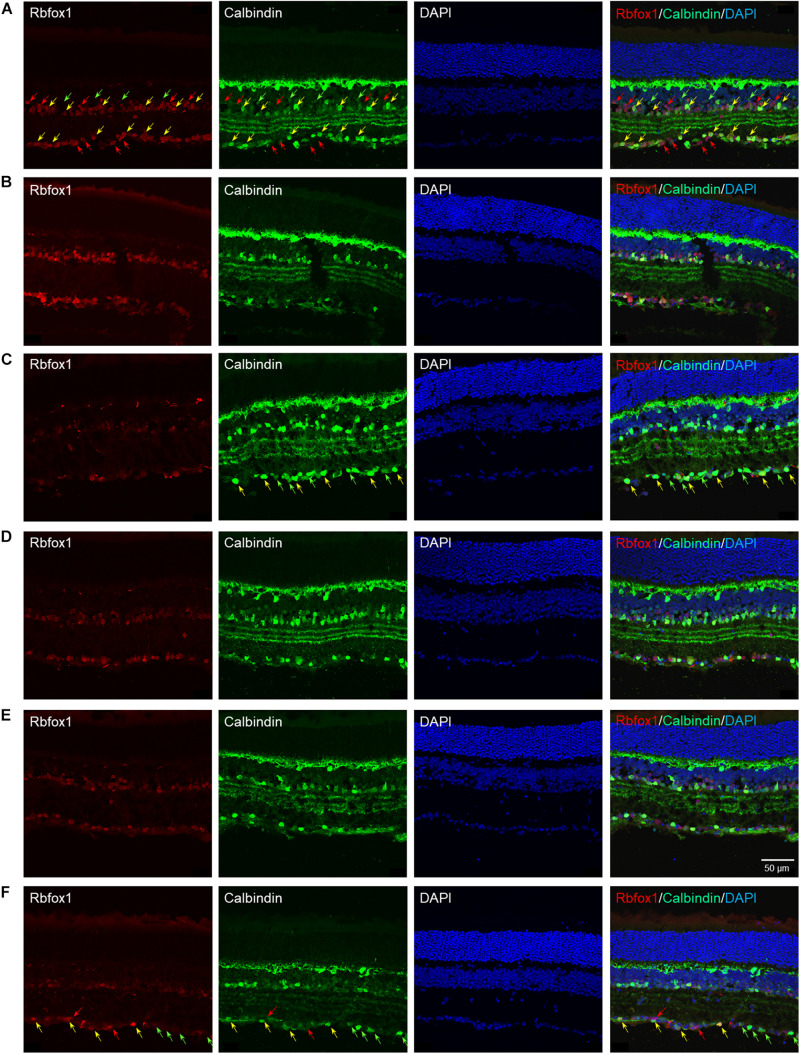
Colocalization of Rbfox1 expression with calbindin-positive ACs. Although, calbindin is an established AC marker, the intensity of calbindin immunoreactivity varies across AC subtypes. Overall, there is a significant overlap between Rbfox1 and calbindin expression within the GCL and innermost layer of INL. No notable difference is observed between calbindin immunoreactivity in control and control treated with tamoxifen retinas **(A,B,D,E)**. Also there no change in calbindin expression pattern in retinas of ONC animals **(A–C)** compared to uninjured groups **(A–C)**. Rbfox1 expression was significantly diminished in *Rbfox1* KO retinas, particularly in the GCL **(C,F)**. Examples of Rbfox1/calbindin-positive cells are indicated by yellow arrows. Rbfox1-positive/calbindin-negative cells in the GCL are pointed with red arrows. Green arrows point at calbindin-positive/Rbfox1-negative cells. Groups was observed when any two groups were compared.

**FIGURE 3:**
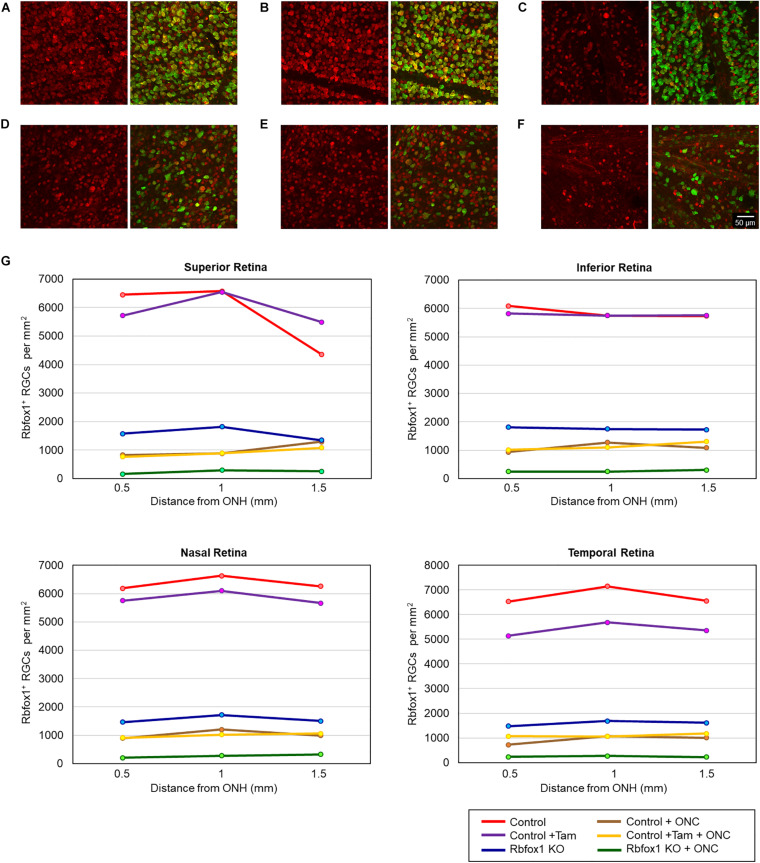
Quantification of Rbfox1-positive RGCs in *Rbfox1*^–/–^ and control animals. Rbfox1/Rbpms-positive cells were counted in the superior, inferior, nasal and temporal retinal quadrants at 0.5, 1, and 1.5 mm from the center of the optic disc. Representative whole mount retinal images of Rbfox1 (left panel) and Rbfox1/Rbpms labeled cells at 1 mm from the center of the optic nerve head from control **(A)**, control + tamoxifen **(B)**, *Rbfox1*^–/–^ **(C)**, control + ONC **(D)**, control + tamoxifen + ONC **(E)**, and *Rbfox1*^–/–^ + ONC **(F)** animals. **(G)** Rbfox1-positive RGC densities in the superior, inferior, nasal and temporal retinal quadrants in both *Rbfox1*^–/–^ uninjured and *Rbfox1*^–/–^ ONC-injured groups were significantly lower compared to that of corresponding control animals.

The extent of Rbfox1 downregulation in *Rbfox1*^*fl/fl*^/UBC-Cre^+/–^ retinas and its effect on retinal morphology was evaluated immunohistochemically with antibodies against Rbfox1, Rbpms (RGC marker) and calbindin (AC marker). Age-matched heterozygous *Rbfox1*^*fl/*+^ animals treated with corn oil (vehicle) or with tamoxifen were used as controls. As expected, Rbfox1 expression in mouse retinas was localized to RGCs and dACs in the ganglion cell layer (GCL) and innermost row of ACs in the inner nuclear layer (INL; Figures 1, 2). Colocalization of the Rbfox1-positive cells with Rbpms-positive cells in retinal sections of heterozygous *Rbfox1*^*fl/*+^ control animals treated with corn oil or with tamoxifen is shown in Figures 1A,B, respectively. No difference between these two control groups was observed, indicating that tamoxifen has no adverse effect on the expression of Rbfox1, Rbpms and calbindin or on retinal morphology (Figures 1, 2). The downregulation of Rbfox1 in retinas of *Rbfox1*^*fl/fl*^/UBC-Cre^+/–^ animals, was more prominent in the GCL and particularly in RGCs. Very few Rbpms/Rbfox1-positive were present (Figure 1C; see below for quantitative data). One week after ONC, the number of Rbpms-positive cells dropped significantly (Figures 1D–F) and almost no Rbfox1 staining was observed in the GCL (Figure 1F; see below for quantitative data). Co-immunostaining with Rbfox1 and calbindin showed significant overlap in the expression of these proteins in ACs localized in the GCL and INL (Figure 2). The vast majority of Rbfox1-positive/calbindin-negative cells in the GCL are RGCs. We also know from our earlier work that some subtypes of dACs (∼7% of dACs) do not express Rbfox1. Dramatic decrease in Rbfox1 expression in dACs in the GCL and ACs in the INL of *Rbfox1* KO animals (Figures 2C,F) compared to control retinas (Figures 2A,B,D,E) was observed. For quantitative analysis of Rbfox1-positive RGCs in *Rbfox1*^–/–^ retinas vs. relevant controls, Rbfox1/Rbpms-immunostained cells were counted in superior, inferior, nasal and temporal retinal quadrants at 0.5, 1, and 1.5 mm from the center of the optic nerve disk in whole mounted retinas (Table 1). The average densities (cells/mm^2^) of Rbfox1-positive RGCs in uninjured animals were: control 6,188 ± 1,155; control + tamoxifen 5,732 ± 810; and *Rbfox1*^–/–^ 1,625 ± 524 (*n* = 6/group; Figures 3A–C,G). In animals with axonal injury, the average numbers of Rbfox1-positive RGCs were: control + ONC 1,016 ± 377; control + tamoxifen + ONC 1,037 ± 439; and *Rbfox1*^–/–^ + ONC 252 ± 173 (*n* = 6/group; Figures 3D–G). This translates to approximately 73 and 82% decrease in the number of Rbfox1-positive RGCs in *Rbfox1*^–/–^ retinas in uninjured and ONC retinas, respectively, compared to that of relevant control groups (*Rbfox1* KO vs. control *P* = 3.53361E-52; *Rbfox1* KO vs. control + tamoxifen *P* = 7.38334E-67; *Rbfox1* KO + ONC vs. control + ONC *P* = 1.4715E-28; *Rbfox1* KO + ONC vs. control + tamoxifen + ONC *P* = 8.01704E-25).

**TABLE 1:** Quantification of Rbfox1-positive RGCs in uninjured and ONC *Rbfox1*^–/–^ and control animals (cells per mm^2^).

Location Group	S 0.5	S 1	S 1.5	I 0.5	I 1	I 1.5	N 0.5	N 1	N 1.5	T 0.5	T 1	T 1.5
Control	6,450949	6,574674	4,3601,465	6,088515	5,746926	5,7351,410	6,189571	6,626894	6,259927	6,531757	7,1471,313	6,5571,286
Control + Tam	5,718870	6,5421,154	5,4951,058	5,819368	5,749557	5,755785	5,755823	6,102679	5,666548	5,1391,001	5,686604	5,359783
Rbfox 1^–/–^	1,577514	1,820504	1,346266	1,814615	1,751625	1,727526	1,467602	1,722551	1,505556	1,476485	1,687468	1,612728
Control + ONC	830190	885410	1,288379	938260	1,273382	1,085362	900221	1,204457	992303	726277	1,065519	1,004474
Control + Tam + ONC	764244	888262	1,076521	1,010330	1,097429	1,302636	914361	1,021553	1,059305	1,071710	1,056397	1,181438
Rbfox1^–/–^ + ONC	159153	292250	255204	243131	243130	298211	205135	281224	324228	22876	272206	220149

**Rbfox1-positive RGCs were counted in superior (S), inferior (I), nasal (N) and temporal (T) retinal quadrants at 0.5, 1, and 1.5 mm from the center of the optic disk*.*

### ONC-Induced RGC Degeneration in Rbfox1 KO Animals

**FIGURE 4:**
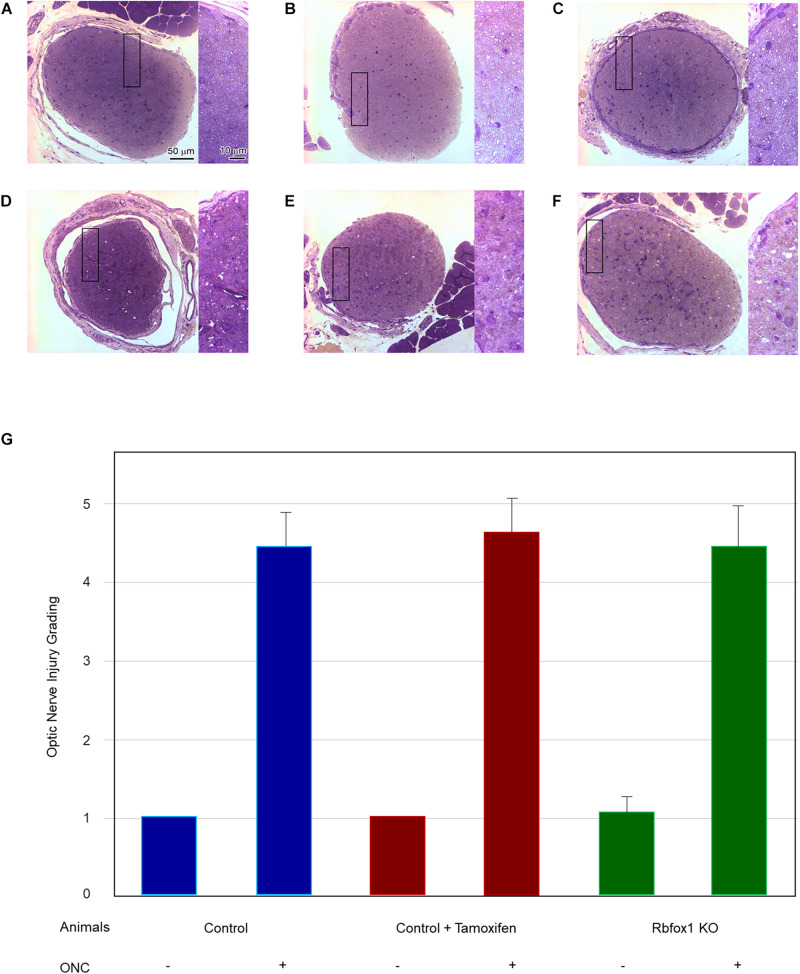
Assessment of axonal degeneration 7 days after ONC. Representative optic nerve micrographs of the uninjured and ONC animals: control **(A)**, control treated with tamoxifen **(B)**, *Rbfox1* KO **(C)**, control with ONC **(D)**, control treated with tamoxifen and ONC **(E)**, Rbfox1 KO with ONC **(F)**. Uninjured optic nerve in all three groups are normal. Optic nerves in the ONC groups sustained severe axonal degeneration, axonal swelling and degeneration of myelin sheath. Rectangular box is presented at higher magnification on the right side of each micrograph. **(G)** Semi-quantitative analysis of optic nerve injury after ONC. The grading of optic nerve in three uninjured groups (no ONC) were scored close to 1. The optic nerve injury grades for ONC groups was ranging from 4.6 to 4.8. No significant difference between these ONC groups was observed when any two groups were compared.

**FIGURE 5:**
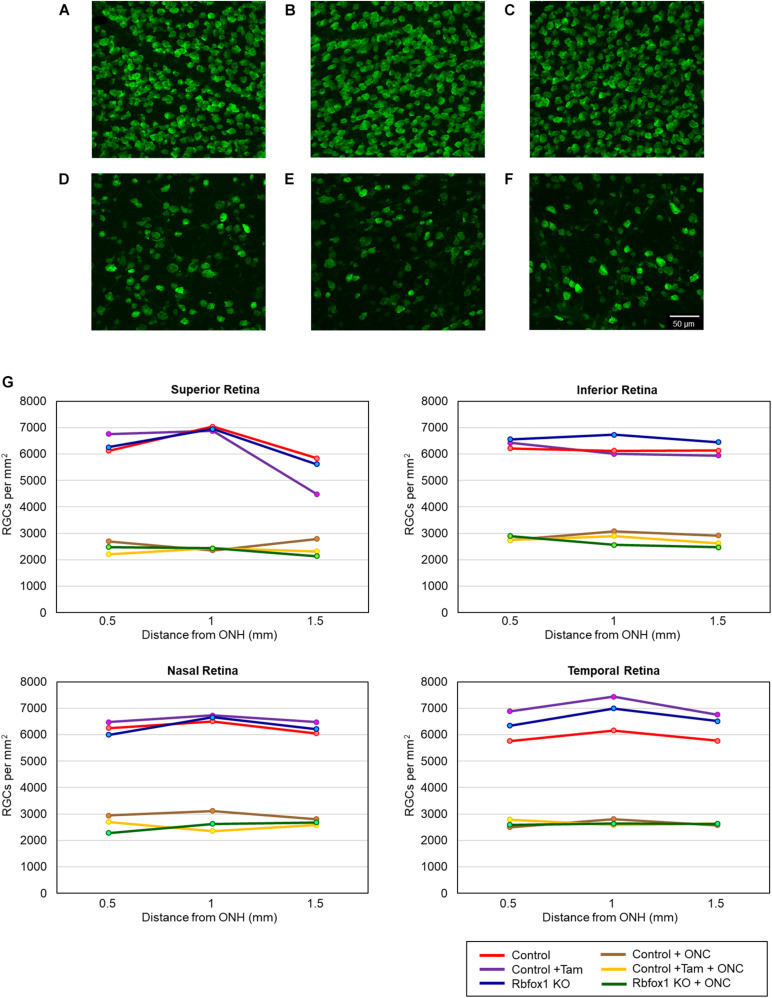
RGC quantification in uninjured and ONC animals. RGCs were counted in the superior, inferior, nasal and temporal retinal quadrants at 0.5, 1, and 1.5 mm from the center of the optic disc. Representative whole mount retinal images of Rbpms labeled RGCs at 1 mm from the center of the optic nerve head from control **(A)**, control + tamoxifen **(B)**, *Rbfox1*^–/–^ **(C)**, control + ONC **(D)**, control + tamoxifen + ONC **(E)**, and *Rbfox1*^–/–^ + ONC **(F)** animals. **(G)** RGC densities in the superior, inferior, nasal and temporal retinal quadrants in all three ONC-injured groups were significantly lower compared to that of corresponding uninjured animals.

**FIGURE 6:**
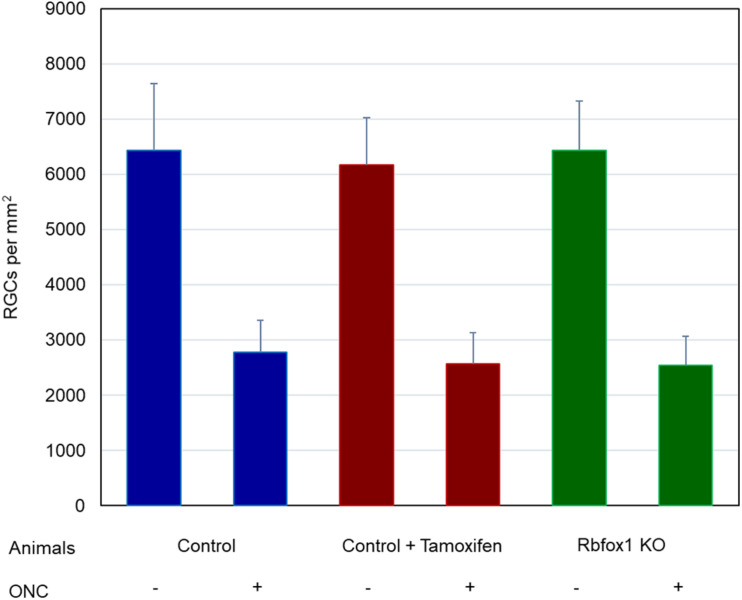
Average RGC densities in *Rbfox1*^–/–^ and control animals. The average RGC densities (cells/mm^2^) were: control 6,438 ± 1,203; control + ONC 2,779 ± 573; control + tamoxifen 6,163 ± 861; control + tamoxifen + ONC 2,573 ± 555; *Rbfox1*^–/–^ 6,437 ± 893; and *Rbfox1*^–/–^ + ONC 2,537 ± 526. The RGC loss in control + ONC, control + tamoxifen + ONC and *Rbfox1*^–/–^ + ONC was 57% (*P* = 1.44954E-42), 58% (*P* = 1.37543E-57) and 61% (*P* = 5.552E-59) compared to RGC numbers in the relevant uninjured groups, respectively. No statistically significant difference was observed between any two groups of uninjured animals or between any two ONC groups.

To evaluate the effect of Rbfox1 deletion on the survival of injured RGCs, six groups of animals were used: control, control/tamoxifen, *Rbfox1*^–/–^, control/ONC, control/tamoxifen/ONC and *Rbfox1*^–/–^/ONC. ONC-induced damage was analyzed by grading the axonal degeneration in the optic nerve and by counting RGC somata in the retina 7 days after injury. Axons in both uninjured control groups, as well as uninjured *Rbfox1* KO animals, appeared to be normal and no noteworthy differences between these groups were observed (Figures 4A–C). Extensive optic nerve degeneration with degenerated myelin sheath, swollen axons and activated glial cells were noted in the optic nerves of all three groups of animals with ONC (Figures 4D–F). Semi-quantitative grading of optic nerve injury in three uninjured groups were scored close to 1 (normal, no degeneration), whereas the average injury grades for groups with ONC ranged from 4.6 to 4.8 (severe degeneration; Figure 4G). Among these three ONC groups, no statistical significance was observed when any two groups were compared. Rbpms-labeled RGC somas were counted in superior, inferior, nasal and temporal retinal quadrants at 0.5, 1, and 1.5 mm from the center of the optic disc. The RGC densities in these locations for all six groups of animals are presented in Table 2 and Figure 5. The average RGC densities (cells/mm^2^) were: control 6,438 ± 1,203; control + ONC 2,779 ± 573; control + tamoxifen 6,163 ± 861; control + tamoxifen + ONC 2,573 ± 555; *Rbfox1*^–/–^ 6,437 ± 893; and *Rbfox1*^–/–^ + ONC 2,537 ± 526 (*n* = 6/group; Figure 6). The RGC loss in control + ONC, control + tamoxifen + ONC and *Rbfox1*^–/–^ + ONC was 57% (*P* = 1.44954E-42), 58% (*P* = 1.37543E-57) and 61% (*P* = 5.552E-59) compared to RGC numbers in the relevant uninjured groups, respectively. No statistically significant difference was observed between any two groups of uninjured animals or between any two ONC groups.

**TABLE 2:** RGC densities in uninjured and ONC *Rbfox1*^–/–^ and control animals (per mm^2^).

Location Group	S 0.5	S 1	S 1.5	I 0.5	I 1	I 1.5	N 0.5	N 1	N 1.5	T 0.5	T 1	T 1.5
Control	6,753956	6,875708	4,4851,529	6,424493	6,007964	5,9431,461	6,476586	6,733969	6,479984	6,884808	7,4391,305	6,7561,330
Control + Tam	6,123912	7,0401,155	5,8391,186	6,212428	6,126458	6,134699	6,2471,006	6,502726	6,050487	5,7611,137	6,160713	5,767939
*Rbfox 1*^–/–^	6,256703	6,947707	5,6191,190	6,554291	6,727511	6,444964	5,992476	6,655428	6,2121,223	6,3371,101	6,994701	6,5101,418
Control + ONC	2,703634	2,352633	2,786530	2,743502	3,076695	2,920627	2,948480	3,119609	2,813534	2,503399	2,813490	2,569703
Control + Tam + ONC	2,208583	2,433547	2,315748	2,746426	2,902468	2,624576	2,700792	2,358544	2,587414	2,795473	2,578466	2,633584
*Rbfox 1*^–/–^ + ONC	2,486392	2,436346	2,135387	2,899611	2,567462	2,471533	2,286416	2,627610	2,679911	2,595565	2,639491	2,627429

**RGCs were counted in superior (S), inferior (I), nasal (N), and temporal (T) retinal quadrants at 0.5, 1, and 1.5 mm from the center of the optic disk.**

## Discussion

Rbfox1 is a moonlighting protein, which regulates alternative splicing, transcription, mRNA stability and translation efficiency. Its role in regulation of gene networks associated with neurogenesis and neuronal function is well established. Recent studies implicate Rbfox1 in the stress-induced regulation of molecular pathways that promote cell survival. A mechanism by which Rbfox1 promotes cell survival during Drosophila oogenesis, which was used as an *in vivo* system for the stress response, involves its interaction with stress-dependent miRNA miR-980 (Kucherenko and Shcherbata, 2018). Stress-induced reduction of miR-980 expression led to an increased level of Rbfox1, extensive formation of ribonucleoprotein (RNP) granules, and resulted in higher cell viability. Rbfox1 was also implicated in the neuroprotective effect of miR-132 against amyloid β-peptide (Aβ) and glutamate excitotoxicity (El Fatimy et al., 2018). miR-132 has been associated with progression of both amyloid and Tau pathology in Alzheimer’s disease. It is the most downregulated miRNA in Alzheimer’s disease brain; the miR-132 downregulation precedes neuronal loss (Smith et al., 2015; Hernandez-Rapp et al., 2016; Salta et al., 2016; Pichler et al., 2017). Neuroprotective effects of miR-132 are mediated by direct regulation of the Tau modifiers including Rbfox1 (El Fatimy et al., 2018). Tau pathology has been also associated with dysfunction and degeneration of RGCs in a rat glaucoma model, 3xTg-AD mouse model of AD and m3R tau-Tg mice exhibiting Pick’s Disease pathology (Chiasseu et al., 2016, 2017; Ngolab et al., 2021). This suggests that the miR-132/Rbfox1-mediated mechanisms that promote neuronal survival in the brain of the AD mouse model may also be present in RGCs to protect these cells from stress-induced damage.

In our earlier work, we have characterized the expression of Rbfox1 in the retina and evaluated the effect of its downregulation on retinal integrity and visual function (Gu et al., 2018). We showed that Rbfox1 expression is restricted to RGCs and predominantly GABAergic ACs in the GCL and INL. Deletion of Rbfox1 in adult mouse retinas had no effect on retinal gross morphology but *Rbfox1*^–/–^ animals exhibited depth perception deficiencies. In this study, we evaluate the survival of RGCs 7 days after ONC in control and *Rbfox1*^–/–^ animals to determine whether or not Rbfox1 is involved in cell protection in response to stress. Downregulation of Rbfox1 in *Rbfox1*^–/–^ is tamoxifen-induced and, although, the Cre-loxP system and its induction by tamoxifen has been established as a reliable and safe method to delete target genes in the retina (Boneva et al., 2016), we have included in this study an additional tamoxifen-treated control group to rule out the potential cytotoxic effect of the drug itself. ONC injury resulted in virtually complete degeneration of RGC axons and more than a twofold decrease in the number of RGC somas. The average RGC densities in uninjured control, control + tamoxifen and *Rbfox1*^–/–^ groups were 6,438, 6,163, and 6,437 per mm^2^, respectively, which well correlate with reported RGC densities in mouse retinas adjacent to the optic disk (Dräger and Olsen, 1981). Average RGC densities as well as RGC densities in any of the four retinal quadrants were similar between *Rbfox1*^–/–^ and control groups indicating that downregulation of Rbfox1 in adult mouse retinas does not undermine RGC integrity. ONC-induced injury resulted in 57, 58, and 61% loss of RGC somas in control + ONC, control + tamoxifen + ONC and *Rbfox1*^–/–^ + ONC animals, respectively. No significant difference in RGC numbers was observed between *Rbfox1*^–/–^ and control groups. Based on these data, we can suggest that Rbfox1-mediated pathways have no effect on promoting RGC survival injured by ONC. Alternatively, since there is a functional redundancy of Rbfox proteins, the loss of Rbfox1 may be compensated by Rbfox2 or Rbfox3. We can also speculate that RGC protective effect in response to the injury is regulated by Rbfox2 or Rbfox3, and not by Rbfox1, as in the above mentioned models of cellular stress (El Fatimy et al., 2018; Kucherenko and Shcherbata, 2018). Rbfox3, for instance, has been shown to be downregulated in ONC-injured RGCs prior to neuronal degeneration (Tran et al., 2019), suggesting that the normal function of this protein is important for RGC survival. Therefore, evaluation of double or triple Rbfox knockouts may provide additional information about the role of Rbfox proteins in supporting the survival of RGCs under stressful conditions.

## Data Availability Statement

The original contributions presented in the study are included in the article/supplementary material, further inquiries can be directed to the corresponding author/s.

## Ethics Statement

The animal study was reviewed and approved by The Animal Research Committee of the University of California at Los Angeles.

## Author Contributions

NP designed the research and wrote the manuscript. LG and JK performed the research. LG, JK, JC, and NP analyzed the data. All authors reviewed the manuscript.

## Conflict of Interest

The authors declare that the research was conducted in the absence of any commercial or financial relationships that could be construed as a potential conflict of interest.
